# Impact of preoperative comorbidities on postoperative complication rate and outcome in surgically resected non-small cell lung cancer patients

**DOI:** 10.1007/s11748-021-01710-5

**Published:** 2021-09-23

**Authors:** Martina Benker, Necati Citak, Thomas Neuer, Isabelle Opitz, Ilhan Inci

**Affiliations:** Department of Thoracic Surgery, Zurich University Hospital, Raemistrasse 100, 8091 Zurich, Switzerland

**Keywords:** NSCLC, Lung resection, Comorbidity, Complication

## Abstract

**Objective:**

This study aimed to analyze whether comorbidities impact postoperative complication rate or survival after anatomical lung resection for non-small cell lung cancer (NSCLC).

**Methods:**

A retrospective analysis of 1219 patients who underwent NSCLC resection between 2000 and 2015 was performed. Analyzed comorbidities included chronic obstructive lung disease (COPD), hypertension, coronary artery disease (CAD), peripheral artery disease, myocardial infarction history, diabetes mellitus, renal insufficiency and other malignancies.

**Results:**

Most patients (78.9%) had comorbidities, most commonly hypertension (34.1%) followed by COPD (26.4%) and other malignancies (19%). The overall complication rate was 38.6% (26.4% pulmonary; 14.8% cardiac; and 3.0% gastrointestinal). Hypertension (odds ratio (OR) = 1.492*, p* = 0.031) was associated with more cardiac complications. Heavy smoking (OR = 1.008, *p* = 0.003) and low body mass index (BMI) (OR = 0.932*, p* < 0.001) affected the pulmonary complication rate significantly. None of the included comorbidities affected the overall complication rate or the survival negatively. However, the patient characteristics of advanced age (*p* < 0.001), low BMI (*p* = 0.008), and low FEV1 (*p* = 0.008) affected the overall complication rate as well as survival (each *p* < 0.001).

**Conclusion:**

Advanced age, low BMI, and low FEV1 were predictive of greater complication risk and shorter long-term survival in patients who underwent NSCLC resection. Cardiac complications were associated with hypertension and CAD, whereas pulmonary complications were associated with a high pack year count.

## Introduction

Lung cancer is a major public health issue worldwide, constituting the second most common cancer in both men and women and being responsible for more cancer-related deaths per year in Switzerland than any other cancer diagnosis [1]. Unfortunately, in more than half of cases, diagnoses of non-small cell lung cancer (NSCLC) are made at an advanced stage and patients with NSCLC tend to have higher comorbidity burdens than patients with other types of malignant tumors, such as breast cancer, prostate cancer, colon cancer, and head and neck cancers [2].

Comorbidity of NSCLC with other tobacco-associated illnesses, including chronic obstructive pulmonary disease (COPD), cardiovascular diseases, and head and neck cancers, can complicate treatment and affect posttreatment survival. Surgical resection remains a critical potentially curative treatment of lung cancer, but having a good outcome is dependent upon the selection of suitable patients for anatomical lung resection of NSCLC.

Population aging has been associated with an increase in the median age of patients being diagnosed with lung cancer, with the age band of patients that are most frequently diagnosed with lung cancer being 60–69 years, followed closely by people that are 70–79 years old [3]. This increase in geriatric patients referred for surgical resection of lung cancer has brought greater attention to how comorbidities should be considered in NSCLC treatment planning [4]. The aim of the present study was thus to analyze whether and how preoperative comorbidities may impact the occurrence of postoperative complications or survival after anatomical lung resection in patients diagnosed with NSCLC.

## Methods

### Patients

This retrospective study examines the cases of patients with a diagnosis of NSCLC who underwent anatomical lung resections between January 2000 and December 2015 at the University Hospital Zurich. Initially, 1′312 such patients were included. Patients with low-frequency characteristics were excluded to facilitate statistical analyses. Specifically, patients under 40 years of age (*N* = 30), patients with a postoperatively determined disease staging of zero (*N* = 12), patients with GOLD stage IV COPD (*N* = 3), and a patient who received only radiotherapy as a preoperative treatment (*N* = 1) were excluded. An additional 47 patients who were lost to follow-up were excluded from the final analysis. After these exclusions, we included a total of 1219 patients. The cantonal ethics committee of Zurich approved this study (2016-00799).

### Comorbidities

The following comorbidities were included in this study: COPD, diabetes mellitus, renal insufficiency, another malignancy; and cardiovascular comorbidities including coronary artery disease (CAD), hypertension, previous myocardial infarction, and peripheral artery disease. Hypertension was defined as a repeated measurement of a blood pressure over 140/90 mmHg. CAD was diagnosed with exercise ECG, myocardial scintigraphy, echocardiography, or coronary angiography. The diagnostic criteria for peripheral artery disease were a high ankle-brachial index, a history of claudication, ischemic rest pain and/or an abnormally low extremity pulse, nonhealing lower extremity wound or gangrene apparent upon physical examination. Post-myocardial infarction status was determined based on chart review and patient history. COPD was defined by a post bronchodilator Tiffenau-Pinelli Index of < 0.7 and divided into four categories according to the GOLD Classification [5]. Diabetes mellitus was included as a comorbidity if diagnosed according to the 1999 World Health Organization guidelines [6]. Any cancer outside the lungs was considered another malignancy. Renal insufficiency was defined as a glomerular filtration rate < 60 ml/min/1.73 m^2^.

### Complications and survival

Postoperative complications were divided into four major groups: cardiac; pulmonary; gastrointestinal; and other (e.g., local and systemic postoperative infections). We included the most common and most severe complications after lung resection (Table 1).

**Table 1:** Postoperative complications

Type of complication Complication diagnosis	Frequency (Percentage)
Pulmonary	
Pneumonia	70 (5.7%)
Prolonged air leak	65 (5.3%)
Pneumothorax	56 (4.6%)
Hematothorax	20 (1.6%)
Empyema	22 (1.6%)
Acute respiratory distress syndrome	17 (1.4%)
Pleural effusion	13 (1.1%)
Bronchopleural fistula	11 (0.9%)
Atelectasis	11 (0.9%)
Chylothorax	9 (0.7%)
Cardiac	
Arrhythmia	149 (12.2%)
Hypertension	29 (2.4%)
Myocardial infarction	3 (0.2%)
Gastrointestinal	
Ileus	9 (0.7%)
Others (Colitis, Ulcer, Bleeding, Ischemia)	27 (2.2%)
Local/Systemic	
Wound infection	26 (2.1%)
Paralysis of recurrent laryngeal nerve	26 (2.1%)
Sepsis	19 (1.6%)
Seroma	18 (1.5%)
Renal insufficiency	13 (1.1%)
Delirium	9 (0.7%)
Aerodermectasia	8 (0.7%)

Pneumonia was defined as bacterial pneumonia diagnosed on chest X-Ray. Atelectasis equally was a radiographic diagnosis. Prolonged air leakage was defined as an air leak that persisted beyond the seventh postoperative day. The diagnosis of ARDS was made in accordance with the Berlin definition of ARDS [7]. Regarding cardiac arrhythmias, both atrial and ventricular arrhythmias were included in this study. Postoperative hypertension was defined as a blood pressure over 140/90 mmHg requiring antihypertensive medication. Sepsis was defined as a life-threatening organ dysfunction caused by a dysregulated response to infection [8]. Postoperative Complications were included within 30 days after surgery.

Operative mortality or 30-day mortality was defined as ‘any death regardless of cause occurring within 30 days after surgery in or out of the hospital, and after 30 days during the same hospitalization subsequent to the operation’ [9]. Furthermore, if a patient had any further surgery during their hospitalization, operative mortality is assigned to the first operation of the given hospitalization.

Follow-up was completed in January of 2019 or upon death.

### Statistical analysis

Statistical analyses were performed in SPSS for Windows version 26.0 (IBM, Armok, NY, USA) and R version 3.6.1 (R Foundation for Statistical Computing, Vienna, Austria). Continuous data are presented as means and standard deviations; categorical data are presented as frequencies and percentages. To identify possible predictive factors, binominal and multivariate logistic regressions were performed to ascertain the effects of patient characteristics and comorbidities on the occurrence of complications. A Cox proportional hazards model was applied to find possible associations of patient characteristics or comorbidities with overall survival. Odds ratios (ORs) and hazard ratios (HRs) are reported as appropriate. In all cases, *p* < 0.05 was considered significant.

## Results

### Patient population, tumor characteristics, and surgery

The demographic characteristics of the patients, their tumor characteristics, and their surgical interventions are summarized for the patient sample as a whole in Table 2, together with comparisons between patients with complications and patients without complications for each variable. With respect to patient characteristics, a majority of the patients were men, most were in their sixties, and a majority had a healthy body mass index (BMI), though about one in twenty was underweight. The mean FEV1 of all subjects was within normal range, and there was a wide range of smoking history in terms of pack years. Notably, tumor histology revealed that resected lung tumors were most frequently adenocarcinomas, followed by squamous cell carcinomas, carcinoids, other tumor types, and large cell carcinomas, respectively. About two thirds of the patients underwent lobectomy, followed in frequency by pneumonectomy and sleeve resection, respectively.

**Table 2:** Patient demographic, tumor, and surgery characteristics of all patients, patients without complications, and patients with complications

Variable category	All patients (*N* = 1219)	Complications		*P*
		None (*N* = 748)	One or more (*N* = 471)	
Age	64.24 (9.53)	63.01 (9.41)	66.20 (9.40)	< 0.001
Gender				0.102
Female	458 (37.6%)	295 (24.2%)	163 (13.4%)	
Male	761 (62.4%)	924 (75.8%)	1056 (86.6%)	
Mean body mass index	25.14 (4.65)	25.32 (4.71)	24.86 (4.54)	0.094
Body mass index category				0.374
Underweight	64 (5.3%)	34 (4.5%)	30 (6.4%)	
Normal	639 (52.4%)	387 (51.7%)	252 (53.5%)	
Overweight	373 (30.6%)	234 (31.3%)	139 (29.5%)	
Obese	143 (11.7%)	93 (12.4%)	50 (10.6%)	
FEV1% predicted	84.17 (20.98)	85.64 (20.73)	81.84 (21.17)	0.002
Forced vital capacity	95.05 (18.92)	96.42 (18.79)	92.88 (18.95)	0.001
Packyears	41.90 (31.05)	40.79 (30.95)	43.66 (31.17)	0.116
Tumor stage				0.135
I	428 (35.1%)	274 (36.6%)	154 (32.7%)	
II	293 (24.0%)	162 (21.7%)	131 (27.8%)	
III	399 (32.8%)	246 (32.9%)	153 (32.5%)	
IV	99 (8.1%)	66 (8.8%)	33 (7.0%)	
Histology diagnosis				0.214
Adenocarcinoma	613 (50.3%)	390 (52.1%)	223 (47.3%)	
Squamous cell carcinoma	382 (31.3%)	225 (30.5%)	157 (33.3%)	
Carcinoid	80 (6.6%)	53 (7.1%)	27 (5.7%)	
Large cell carcinoma	60 (4.9%)	31 (4.1%)	29 (6.2%)	
Other	84 (6.9%)	49 (6.6%)	35 (7.4%)	
Neoadjuvant treatment				0.235
None	1005 (82.4%)	609 (81.4%)	396 (84.1%)	
Chemotherapy	176 (14.4%)	111 (14.8%)	65 (13.8%)	
Radiotherapy and chemotherapy	38 (3.1%)	28 (3.7%)	10 (2.1%)	
Surgery				0.076
Open	839 (68.8%)	508 (67.9%)	331 (70.3%)	
Minimally invasive	179 (14.7%)	123 (16.4%)	56 (11.9%)	
Conversion	201 (16.5%)	117 (15.6%)	84 (17.8%)	
Surgery type				0.437
Lobectomy	819 (67.2%)	509 (68.0%)	310 (65.8%)	
Sleeve resection	181 (14.8%)	113 (15.1%)	68 (14.4%)	
Pneumonectomy	219 (18.0%)	126 (16.8%)	93 (19.7%)	

Data are in the form of mean (standard deviation) or frequency (percentage)

### Overall comorbidities and complications

**Fig. 1:**
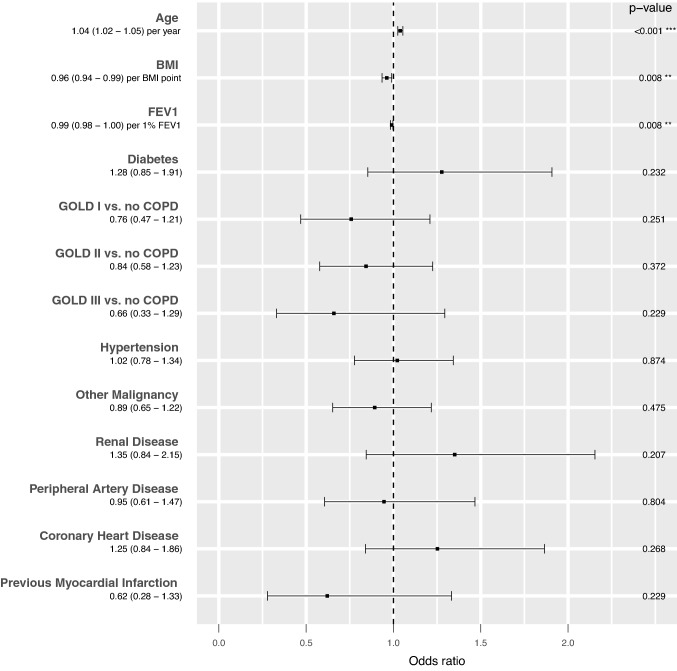
Forest plot depicting the impacts of patient characteristics and preoperative comorbidities on the overall occurrence of postoperative complications. A more advanced age, lower BMI, and lower FEV1 were significantly associated with a greater complication rate

A large majority of the patient sample (*N* = 957, 78.9%) had at least one identified comorbidity. The overall postoperative complication rate was 38.6% (*N* = 471). The occurrence of comorbidities overall and comparisons of the occurrence of complications in patients with versus without each analyzed comorbidity are reported in Table 3. According to our multivariate logistic regression analysis performed to detect independent influencing factors on postoperative complication rate, none of the analyzed comorbidities was a significant influencing factor. However, overall complication risk was influenced by some patient characteristics. Specifically, as shown in Fig. 1, complication risk was found to be positively associated with older age (*p* < 0.001), lower BMI (*p* = 0.008), and lower FEV1 (*p* = 0.008).

**Table 3:** Associations of comorbidities, by type and by specific diagnosis, with the occurrence of complications in patients following anatomical Non-small cell lung cancer tumor resection

Type of comorbidity Comorbidity diagnosis category	Overall	Complications		*P*
		None (*N* = 748)	One or more (*N* = 471)	
Pulmonary				
Diagnosis of chronic obstructive pulmonary disease (any stage)	322 (26.4%)	191 (25.5%)	131 (27.8%)	0.417
Chronic obstructive pulmonary disease GOLD stage				0.61
I	91 (7.5%)	58 (7.8%)	33 (7.0%)	
II	181 (14.8%)	104 (13.9%)	77 (16.3%)	
III	50 (4.1%)	29 (3.9%)	21 (4.5%)	
Cardiovascular				
Arterial hypertension	416 (34.1%)	243 (32.5%)	173 (36.7%)	0.144
Coronary heart disease	155 (12.7%)	83 (11.1%)	72 (15.3%)	0.04
Peripheral artery disease	104 (8.5%)	57 (7.6%)	47 (10.0%)	0.184
Myocardial infarction history	35 (2.9%)	22 (2.9%)	13 (2.8%)	0.993
Other comorbidity				
Diabetes mellitus	129 (10.6%)	70 (9.4%)	59 (12.5%)	0.098
Renal insufficiency	90 (7.4%)	47 (6.3%)	43 (9.1%)	0.082
Other malignancy	232 (19%)	145 (19.4%)	87 (18.5%)	0.748

Furthermore, there was no multicollinearity among variables, assuring the independence of each influencing factor included in this multivariate logistic regression.

### Pulmonary complications

Two factors were found to be significant predictors of a higher rate of pulmonary complications, namely a low (underweight) BMI (*p* < 0.001) and a high pack year smoking history (*p* = 0.003). A low FEV1 had a non-significant trend toward being inversely related to the incidence of pulmonary complications (*p* = 0.065). Surprisingly, COPD was not found to be related to postoperative pulmonary complication risk (*p* = 0.525). Despite increased age being a negative prognostic factor for survival and the overall occurrence of complications, it was not associated with a higher pulmonary complication rate specifically (*p* = 0.619).

### Cardiovascular complications

Patients with arterial hypertension (*p* = 0.031) and older patients (*p* < 0.001) were found to be at significantly elevated risk of postoperative cardiac complications. Pneumonectomy was identified as an independent risk factor for cardiac complications (*p* = 0.016). Whereas the cardiovascular comorbidities of CAD and peripheral artery disease did not have significant influences on the occurrence of cardiac complications (*p* = 0.096 and *p* = 0.225), patients with a history of myocardial infarction appeared to be potentially at a reduced risk of cardiac complications (OR = 0.283). However, there was only a near-significant trend (*p* = 0.052) and the group with a history of myocardial infarction was small (*N* = 35).

### Gastrointestinal complications

The incidence of gastrointestinal complications was very low. Only 36 (3.0%) of the 1219 patients were affected, including 9 patients who suffered from postoperative ileus. Other gastrointestinal complications included bleeding, ischemia, and colitis. No further analysis in regard to predictive factors was feasible given the small number of patients affected.

### Survival

Cox proportional-hazard analysis showed that increased age (HR = 1.02, confidence interval (CI) = 1.01–1.03), lower BMI (HR = 0.96, CI = 0.94–0.98), and lower FEV1 (HR = 0.99, CI = 0.98–0.99) were independent negative influencing factors on overall survival (all *p* < 0.001). Female patients showed a better survival than their male counterparts (HR = 0.77, CI = 0.63–0.94, *p* = 0.009). COPD GOLD stage I (HR = 0.70, CI = 0.49–1.00, *p* = 0.051) and II (HR = 0.73, CI = 0.57–0.95, *p* = 0.019) were associated with a longer overall survival. The group of patients with COPD GOLD III likewise had a lower HR (0.76, CI = 0.50–1.15), but this finding was not significant (*p* = 0.191). All other tested comorbidities did not have a significant influence on overall survival.

**Fig. 2:**
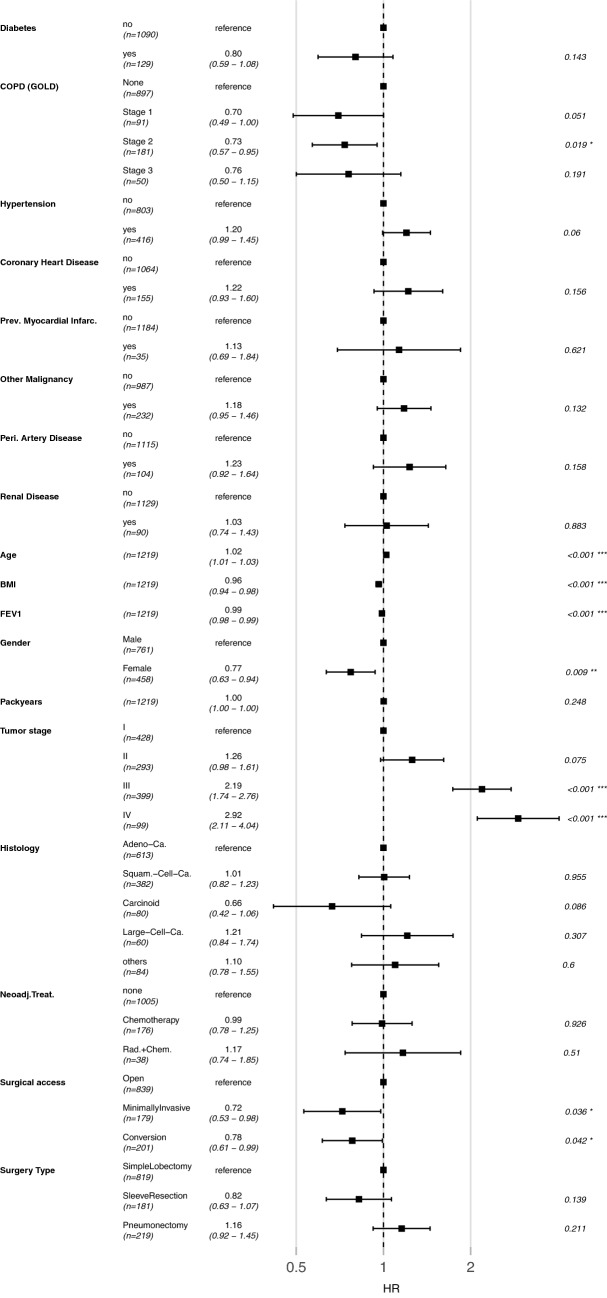
Cox proportional-hazard analysis showing independent influencing factors on survival. Significant factors are indicated with *p < 0.05, **p < 0.01, and ***p < 0.001

Because tumor and surgical characteristics are well-established factors that affect survival, they were included in the Cox proportional-hazard analysis as confounding factors. As expected, the analysis showed that higher tumor stage and an open surgical access were negative prognostic factors for survival. All variables that were included in the Cox proportional-hazard model are shown in Fig. 2.

### Operative mortality

The overall surgical mortality was 1.56% (*n* = 19). The biggest group were patients who died due to multi-organ failure caused by sepsis (*n* = 9). Five patients died postoperatively as a result of ARDS. Two patients suffered massive pulmonary embolisms which led to death. Another two patients died due to substantial postoperative myocardial infarctions. One patient died after a bilateral thromboembolic occlusion of the internal carotid artery.

## Discussion

At the time of NSCLC diagnosis, many patients have serious comorbidities. The high frequency of comorbidities is mainly due to the common risk factor of an extensive history of tobacco use. Furthermore, the median age of patients with lung cancer is around 70 years, which increases the likelihood of pre-existing age-related disease like renal insufficiency or cardiac disease [10]. For these reasons, it is critical to understand the impact of comorbidities on postoperative complications and survival after NSCLC tumor resection.

In the present study, we found that the prevalence of serious comorbidity in patients with NSCLC was quite high, especially in elderly patients.

Although there is a widespread belief amongst clinicians that patients with comorbidities are at an increased risk of postoperative complications, we found that some patient characteristics were more predictive of postoperative problems than any specific comorbidity. In particular, complication risk was found to be significantly associated with older age, a low BMI, and a low preoperative FEV1, affirming the findings of several previous studies [11–13].

None of the analyzed comorbidities was found to be an independent predictor of whether or not a patient would suffer complications overall. However, looking at system specific comorbidities and complications, some correlations could be found. Pulmonary complications were the most frequent complications after lung resections. Similar to a study from Im et al., our analysis indicated that patients with a low BMI and patients with a high pack year count did have a higher rates of pulmonary complications [11]. The effect of smoking as a prognostic factor for pulmonary complications, independent of lung function, can be explained by ciliary damage that impedes mucus clearance, which in turn puts patients at risk of pneumonia [14]. However, none of the analyzed comorbidities—not even COPD—affected the incidence of pulmonary complications significantly.

The most prevalent cardiac complication was atrial arrhythmia, followed by postoperative hypertension; only three patients suffered from a postoperative myocardial infarction. A vast variety of risk factors for atrial fibrillation after pulmonary resection has been reported [15–17]. The present observations of arterial hypertension, higher age, and pneumonectomy being found to be significant independent prognostic factors of a higher cardiac complication rate are not surprising given that hypertension leads to hypertensive heart disease and thus to architectural changes in the myocardium that leave it more susceptible to the development of arrhythmias [18]. The manipulation of the pulmonary veins during pneumonectomy may trigger arrhythmias after this type of surgery [15].

Although patients with CAD had a higher rate of cardiac complications overall, the presence of CAD as a comorbidity was not found to be a significant influencing factor in our multivariate logistic regression (*p* = 0.268). Moreover, because patients with CAD generally have arterial hypertension, it is likely that systemic hypertension was a confounding factor. There are conflicting reports regarding the influence of peripheral vascular disease on postoperative complication [17, 19]. However, in this study, the presence of peripheral artery disease did not influence the occurrence of postoperative cardiac complications. Our finding suggestive of a lower incidence of cardiac complications among patients with a history of myocardial infarction should be considered with caution given that there were only 35 patients in this comorbidity group. It is possible that these patients were assessed more carefully preoperatively or protected by medications they were already taking. Notwithstanding, it would be of interest to examine whether this association would be reproduced in a larger study population.

Many patients who have NSCLC suffer from severe comorbidities that themselves have a direct negative impact on survival and that may be a counter-indication for the use of preferred antineoplastic therapies. Our findings of better survival among COPD patients (significant for GOLD I and II patients) than among patients without COPD contrasts with previous data [20]. There is no clear explanation for these results, though it is possible that other underlying disease not included in our analysis may have affected the results. No other comorbidities included in this study were found to influence survival. Although the presently obtained slightly elevated HR for hypertension was not significant, one would expect that arterial hypertension would be associated with a shorter overall survival, especially if not well managed. It would be of interest in the future to track disease free survival in addition to, or instead of, overall survival.

Our overall survival data indicate that patient characteristics are more important predictors of postoperative survival among NSCLC patients than comorbidities. Increased age, a low BMI, and a low preoperative FEV1 were independent prognostic factors found to be negatively related to overall survival, consistent with previous studies [21, 22].

The present analysis showed that a more advanced age was associated with both a higher complication rate and reduced overall survival. Age-related risks include changes to rib cage calcification, reduced vertebral height due to osteoporosis, and overall deterioration of the immune response leading to a higher susceptibility to infection [23, 24]. The aforementioned anatomical changes result in lower chest wall compliance and reduced inspiratory expansion. Older patients are also more likely to suffer from heart disease and compromised kidney function. However, our multivariate analysis showed age to be an independent influencing factor, while comorbidities per se were mostly not significantly related to postoperative outcome. Hence, it appears that the influence of age on complication rate involves factors not explicitly included in the analysis and, thus, not measured in routine preoperative evaluations [25].

Male sex was an independent, negative prognostic factor for survival. Given that we tracked overall survival and not disease-free survival, this effect could be consequent to women living longer in general. Several hypotheses have been proposed to explain the longevity of women, from the influence of estrogen to the role of mitochondria [26, 27].

The operative mortality in this dataset was 1.56%. This result is at the lower end of the ranges observed by other authors [28, 29].

## Conclusion

In the present study, increased age, a low BMI, and a low FEV1 were independently associated with a higher risk of postoperative complications and shorter survival in NSCLC patients who have undergone anatomical lung resections. Although older patients should not be excluded from surgery based on age alone, their BMI and FEV1 should be considered in the preoperative assessment.

Comorbidities were common amongst patients undergoing anatomical lung resection for NSCLC. However, almost all of them were not found to have a major effect on complications or survival. Only cardiac complications were found to occur more frequently in patients already suffering from arterial hypertension. In summary, this study showed that none of the included comorbidities alone should be an exclusion criterion for surgical treatment for NSCLC.

## Abbreviations

NSCLC: Non-small cell lung cancer
FEV1: 1-S forced expiratory volume
COPD: Chronic obstructive pulmonary disease
ECG: Electrocardiogram
CAD: Coronary artery disease
SPSS: Statistical Package for Social Sciences
OR: Odds ratio
HR: Hazard ratio
BMI: Body mass index
CI: Confidence interval
